# Specific Plasma MicroRNA Signatures Underlying the Clinical Outcomes of Hepatitis E Virus Infection

**DOI:** 10.1128/spectrum.04664-22

**Published:** 2023-01-25

**Authors:** Maria I. Costafreda, Silvia Sauleda, Mar Riveiro-Barciela, Angie Rico, Meritxell Llorens-Revull, Susana Guix, Rosa M. Pintó, Albert Bosch, Francisco Rodríguez-Frías, Ariadna Rando, Maria Piron, Marta Bes

**Affiliations:** a Blood and Tissue Bank of Catalonia (Banc de Sang i Teixits de Catalunya), Transfusion Safety Laboratory, Barcelona, Spain; b Centro de Investigación Biomédica en Red de Enfermedades Hepáticas y Digestivas (CIBEREhd), Instituto de Salud Carlos III, Madrid, Spain; c Vall d’Hebron Institute of Research (VHIR), Vall d’Hebron Universitary Hospital, Barcelona, Spain; d Enteric Virus Laboratory, Department of Genetics, Microbiology and Statistics, School of Biology, and Institute of Nutrition and Safety, University of Barcelona, Barcelona, Spain; e Liver Unit, Internal Medicine Department, Vall d’Hebron Universitary Hospital and Universitat Autonoma de Barcelona, Barcelona, Spain; f Liver Pathology Laboratory, Microbiology and Biochemistry Department, Vall d’Hebron Clinical Laboratories, Vall d’Hebron Universitary Hospital, Barcelona, Spain; Quest Diagnostics

**Keywords:** diagnostics, hepatitis E virus, infection outcomes, microRNA, prognostic indicators

## Abstract

The pathogenic mechanisms determining the diverse clinical outcomes of HEV infection (e.g., self-limiting versus chronic or symptomatic versus asymptomatic) are not yet understood. Because specific microRNA signatures during viral infection inform the cellular processes involved in virus replication and pathogenesis, we investigated plasma microRNA profiles in 44 subjects, including patients with symptomatic acute (AHE, *n* = 7) and chronic (CHE, *n* = 6) hepatitis E, blood donors with asymptomatic infection (HEV BDs, *n* = 9), and anti-HEV IgG^+^ IgM^−^ (exposed BDs, *n* = 10) and anti-HEV IgG^−^ IgM^−^ (naive BDs, *n* = 12) healthy blood donors. By measuring the abundance of 179 microRNAs in AHE patients and naive BDs by reverse transcription-quantitative PCR (RT-qPCR), we identified 51 potential HEV-regulated microRNAs (*P* value adjusted for multiple testing by the Benjamini-Hochberg correction [*P*_BH_] < 0.05). Further analysis showed that HEV genotype 3 infection is associated with miR-122, miR-194, miR-885, and miR-30a upregulation and miR-221, miR-223, and miR-27a downregulation. AHE patients showed significantly higher levels of miR-122 and miR-194 and lower levels of miR-221, miR-27a, and miR-335 than HEV BDs. This specific microRNA signature in AHE could promote virus replication and reduce antiviral immune responses, contributing to the development of clinical symptoms. We found that miR-194, miR-335, and miR-221 can discriminate between asymptomatic HEV infections and those developing acute symptoms, whereas miR-335 correctly classifies AHE and CHE patients. Our data suggest that diverse outcomes of HEV infection result from different HEV-induced microRNA dysregulations. The specific microRNA signatures described offer novel information that may serve to develop biomarkers of HEV infection outcomes and improve our understanding of HEV pathogenesis, which may facilitate the identification of antiviral targets.

**IMPORTANCE** There is increasing evidence that viruses dysregulate the expression and/or secretion of microRNAs to promote viral replication, immune evasion, and pathogenesis. In this study, we evaluated the change in microRNA abundance in patients with acute or chronic HEV infection and asymptomatic HEV-infected blood donors. Our results suggest that different HEV-induced microRNA dysregulations may contribute to the diverse clinical manifestations of HEV infection. The specific microRNA signatures identified in this study hold potential as predictive markers of HEV infection outcomes, which would improve the clinical management of hepatitis E patients, particularly of those developing severe symptoms or chronic infections. Furthermore, this study provides new insights into HEV pathogenesis that may serve to identify antiviral targets, which would have a major impact because no effective treatments are yet available.

## INTRODUCTION

Hepatitis E virus (HEV) belongs to the genus *Orthohepevirus* within the *Hepeviridae* family (1) and is the most common cause of acute viral hepatitis worldwide (2). This virus causes an estimated 20 million infections annually, resulting in 3.4 million symptomatic hepatitis E cases and 70,000 deaths (3). Among the four HEV genotypes infecting humans, genotypes 1 and 2 cause large outbreaks and epidemics in developing countries (3), where HEV causes up to 30% mortality in pregnant women in the third trimester of gestation (4, 5). In contrast, genotypes 3 and 4 are associated with zoonotic infections, which are becoming an emerging threat in industrialized countries given the widespread prevalence of the virus among humans and animal reservoirs (2, 6). Although these infections are typically asymptomatic, chronic infections have been reported among immunocompromised patients, who are at risk of developing cirrhosis, acute decompensation, and liver failure (7, 8). HEV may also cause extrahepatic manifestations including neurological disorders such as Guillain-Barré syndrome, neuralgic amyotrophy, inflammatory polyradiculopathy, bilateral brachial neuritis, encephalitis, myelitis, necrotizing myositis, and multifocal neuropathy, kidney damage, pancreatitis, and hematological complications (8, 9). Notably, a French study reported neurological symptoms in 16.5% of symptomatic HEV infections, and these were more frequent among immunocompetent patients (10). However, host and viral factors determining the development of hepatic and extrahepatic symptoms or the establishment of persistent infections are incompletely understood. Further, no effective treatments are available yet, and the off-label use of ribavirin can result in selection of resistant mutants (11).

Several microRNAs (miRNAs) have been found to regulate both biological and pathological liver processes (12–14). These single-stranded noncoding RNA molecules of 20 to 24 nucleotides bind to complementary sequences in the 3′ untranslated region of mRNAs to control their translation, thereby regulating a wide range of cellular functions, including proliferation, differentiation, and apoptosis (15). In turn, viruses may use cellular and viral miRNAs to promote viral replication, immune evasion, and pathogenesis (16–18). There is increasing evidence that viral infections alter microRNA expression and/or secretion, which may lead to changes in the abundance of extracellular microRNAs. For instance, hepatitis C virus (HCV) infection is associated with increased serum levels of several microRNAs including miR-20a, miR-92a, miR-122, miR-885-5p, miR-134, miR-320c, and miR-483-5p (19–21). Furthermore, the levels of circulating miR-122 and miR-20a, which remain elevated in persistent HCV infection, correlate with HCV-induced liver inflammation and fibrosis progression, respectively (20–22). Although changes in microRNA expression patterns caused by HEV infection have been less extensively characterized than those of HCV infection, specific microRNA signatures have been associated with HEV-induced acute liver failure in pregnant women (23), and serum microRNA profiling has shown potential for the diagnosis of chronic hepatitis E (CHE) (24). However, there is still limited information on dysregulation of microRNAs in HEV infection. Thus, we aimed to study the circulating microRNA profiles during subclinical HEV infections as well as symptomatic acute and chronic HEV infections in our area, where HEV genotype 3 is the predominant genotype. Our findings demonstrated that diverse clinical outcomes of HEV infection are associated with specific microRNA signatures, which can influence disease severity by modulating antiviral immune responses and virus replication.

## RESULTS

### HEV infection alters the circulating levels of microRNAs.

**FIG 1:**
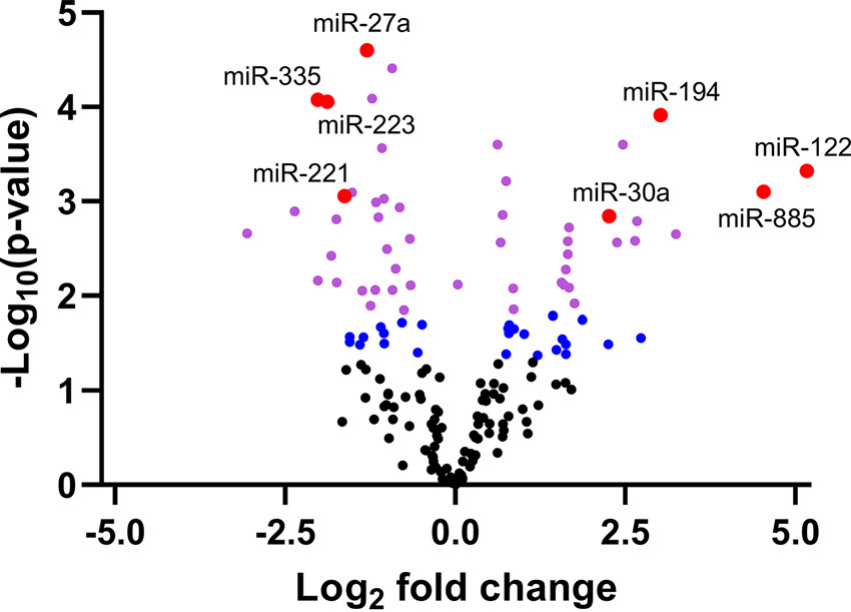
HEV infection is associated with an altered plasma microRNA profile. The volcano plot shows the relationship between the *P* values and the dd*C_q_*. Blue spots correspond to microRNAs with *P* values of <0.05 that did not pass the Benjamini-Hochberg correction for multiple testing (*P*_BH_ > 0.05). Purple and red spots correspond to microRNAs with *P*_BH_ values of <0.05. Large red spots represent the microRNAs that were further investigated in this study.

MicroRNA profiling in plasma samples of acute hepatitis E (AHE) patients and naive blood donors (BDs) revealed that 76 miRNAs were differentially expressed (*P* < 0.05) during acute HEV infection. Of these, 51 passed the Benjamini-Hochberg correction at a significance level of 0.05 (Fig. 1 and see Table S1 in the supplemental material). Notably, the unsupervised analysis showed that the samples clustered according to their biological groups, indicating that HEV infection was causing the largest variation between the samples from AHE patients and naive BDs (Fig. S1).

The plasma levels of miR-122, a liver-specific microRNA (25), and miR-885 were significantly increased in AHE patients compared with naive BDs by an average of 35.9-fold (*P* value adjusted for multiple testing by the Benjamini-Hochberg correction [*P*_BH_] = 0.008) and 23.1-fold (*P*_BH_ = 0.01), respectively. Other microRNAs that were more abundant in plasma of AHE patients than in that of naive BDs include miR-194 (8.1-fold; Mann-Whitney test [MW] *P*_BH_ = 0.02), miR-155 (5.5-fold; *P*_BH_ = 0.005), and miR-30a (4.8-fold; *P*_BH_ = 0.012). In contrast, significantly reduced levels of miR-335 (−4.1-fold; MW *P*_BH_ = 0.02), miR-223 (−3.7-fold; *P*_BH_ = 0.003), miR-221 (−3.1-fold; *P*_BH_ = 0.01), miR-27a (−2.5-fold; *P*_BH_ = 0.003), and miR-23a (−1.9-fold; *P*_BH_ = 0.003) among others, were observed in plasma of AHE patients compared with naive BDs.

### The different outcomes of HEV infection are associated with specific microRNA dysregulations.

The circulating microRNA signature for HEV infection was further validated by comparing the expression levels of the above-mentioned microRNAs in CHE and AHE patients, HEV BDs, exposed BDs, and naive BDs. Only miR-155 was not included in the validation study because it was undetectable in 33.3% of AHE patients (2 of 6) and 33.3% of naive BDs (2 of 6) included in the microRNA profiling assay.

**FIG 2:**
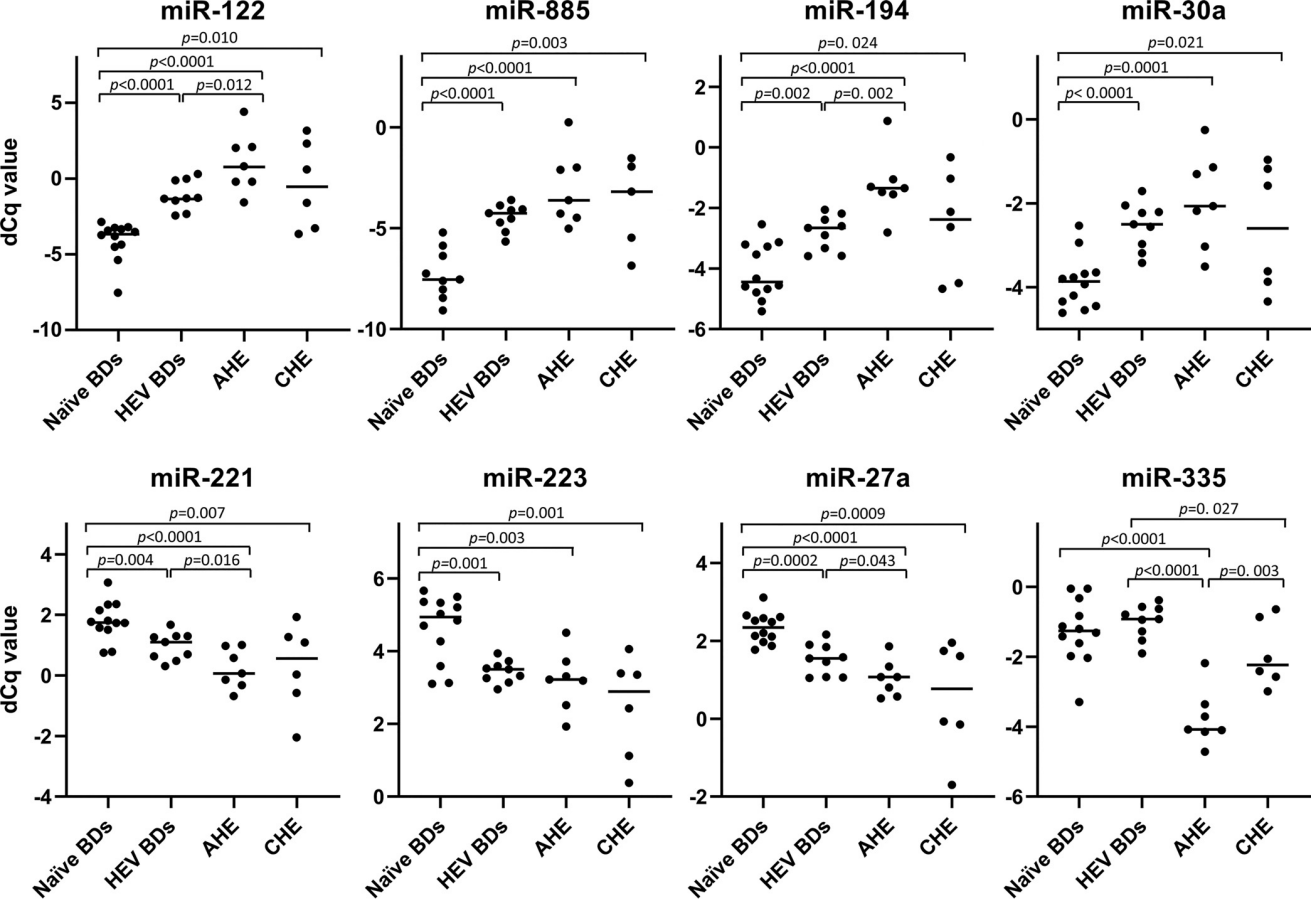
Specific microRNA dysregulations during HEV infection. Scatterplots were constructed with normalized *C_q_* values of the indicated microRNAs in plasma of naive BDs (*n* = 12), AHE patients (*n* = 7), HEV BDs (*n* = 9), and CHE patients (*n* = 6). Upper panels correspond to upregulated microRNAs (miR-122, miR-885, miR-194, and miR-30a). Lower panels correspond to downregulated microRNAs (miR-221, miR-223, miR-27a, and miR-335). Each dot represents an individual sample. *P* values between groups were analyzed by unpaired two-tailed *t* test for normally distributed data or two-tailed Mann-Whitney test (MW) for nonparametric data. *P* values of <0.05 were considered significant.

The validation study confirmed that 7 of 8 analyzed microRNAs were differentially expressed in AHE patients, HEV BDs, and CHE patients compared with naive BDs (Fig. 2). In all HEV-infected groups, miR-122, miR-885, miR-194, and miR-30a were upregulated compared with the naive BD group. In contrast, miR-221, miR-223, and miR-27a were downregulated in all HEV-infected groups compared with naive BDs.

Interestingly, miR-335 was downregulated in AHE patients, but not in HEV BDs or CHE patients, compared with naive BDs (Fig. 2). Additionally, the circulating levels of miR-335 were significantly higher in HEV BDs than in CHE patients.

On the other hand, miR-122, miR-194, miR-335, miR-221, and miR-27a were differentially expressed in AHE patients compared with HEV BDs (Fig. 2). Whereas miR-122 and miR-194 were upregulated in AHE patients, miR-335, miR-221, and miR-27a were downregulated in AHE patients compared with HEV BDs.

No differences were found between naive and exposed BDs (Fig. S2).

### Circulating miR-122 levels correlate with liver damage and viral replication.

**FIG 3:**
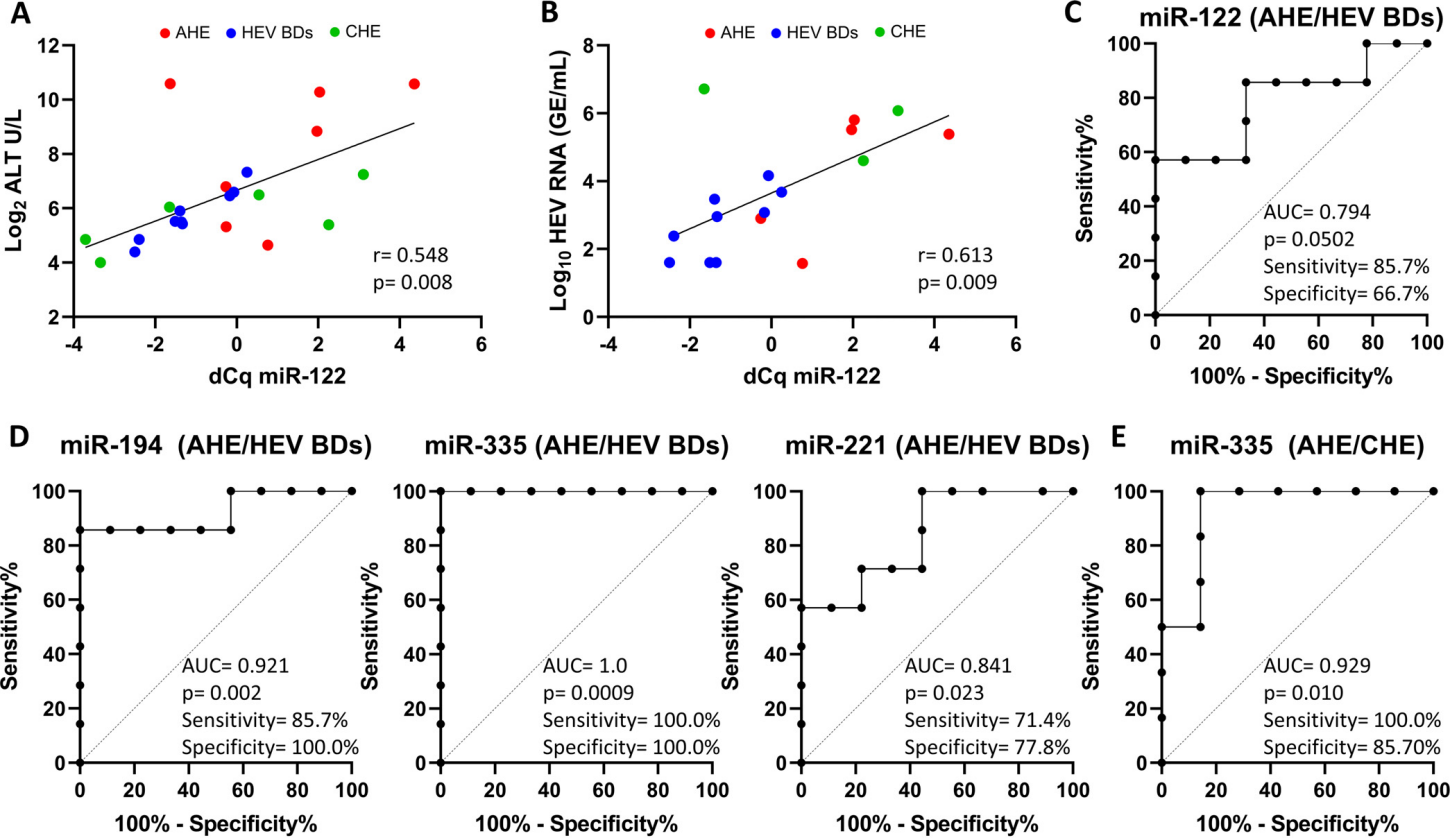
Predictive performance of circulating microRNA levels in diverse HEV infection outcomes. (A and B) Correlation between miR-122 levels and ALT (A) or viral load (B). GE, genome equivalents. The degree of association between miR-122 and ALT and between miR-122 and viral load was calculated by Spearman rank correlation and Pearson correlation coefficients, respectively. Correlation coefficients (*r*) and *P* values are indicated in the graphs. (C and D) ROC plot analysis of predictive discrimination of AHE patients from HEV BDs by miR-122 (C), miR-194 (D, left panel), miR-335 (D, center panel), and miR-221 (D, right panel). (E) ROC plot analysis of predictive discrimination of CHE from AHE patients by miR-335. AUC, *P* value, and sensitivity and specificity values are indicated. *P* values of <0.05 were considered significant. Sensitivity and specificity values are given based on the best compromise between these parameters.

Because miR-122 levels were previously associated with liver injury in different types of liver disease (20, 24, 26–28), we assessed whether miR-122 was associated with liver damage by correlating the circulating miR-122 levels with alanine transaminase (ALT). Whereas a positive correlation was found between these two parameters (*r* = 0.548; *P* = 0.008) (Fig. 3A), no correlation was observed between ALT levels and the abundance of miR-let-7i-5p (*r* = 0.312; *P* = 0.158), which was used as a control (Fig. S3A). We further established a positive correlation between miR-122 and viral load (*r* = 0.613; *P* = 0.009) (Fig. 3B), while no such correlation was found with miR-let-7i-5p (*r* = 0.019; *P* = 0.598) (Fig. S3A). These results indicated that miR-122 levels were associated with both liver damage and HEV replication.

### Performance of circulating microRNAs for predicting the clinical outcome of HEV infection.

To assess the predictive performance of investigated microRNAs for discriminating AHE patients from HEV BDs or CHE patients, we performed a receiver operating characteristic (ROC) analysis. Whereas the circulating levels of miR-122 (area under the curve [AUC] = 0.794; *P* = 0.0502) narrowly failed to achieve a significant predictive value (Fig. 3C), miR-194 (AUC = 0.921; *P* = 0.002), miR-335 (AUC = 1.0; *P* = 0.0009), and miR-221 (AUC = 0.841; *P* = 0.023) correctly discriminated subclinical HEV infections in HEV BDs from symptomatic HEV infections in AHE patients with 71.4% to 100.0% sensitivity and 77.8% to 100.0% specificity (Fig. 3D). In contrast, the circulating levels of miR-885, miR-223, miR-27a, and miR-30a did not show significant predictive value (Fig. S4). With 100.0% sensitivity and 100.0% specificity, circulating miR-335 levels showed the best predictive performance for identifying acute symptomatic HEV infections. In addition, miR-335 levels (AUC = 0.929; *P* = 0.010) were also capable of predicting chronic HEV compared with AHE patients with 100.0% sensitivity and 85.7% specificity (Fig. 3E).

## DISCUSSION

According to the latest global estimation, there are approximately 15 million individuals with an ongoing HEV infection (29). The infection is often asymptomatic, but severe clinical outcomes can develop, especially in pregnant women and immunosuppressed patients. Despite the significant burden of HEV infection, the pathophysiological mechanisms underlying the different prognoses are not yet understood.

Given the importance of microRNAs in virus replication and pathogenesis (see reference 30 and references therein), we studied the relationship between specific plasma microRNA signatures and both symptomatic and asymptomatic acute HEV infections, as well as chronic HEV infections. Initial profiling of circulating microRNAs in plasma of naive BDs and AHE patients showed significant differences in the abundance of 51 microRNAs after applying the Benjamini-Hochberg correction for multiple testing, indicating that HEV infection alters the expression or secretion of these microRNAs. Among them, miR-122, miR-194, miR-221, miR-223, miR-27a, miR-30a, miR-335, and miR-885 were selected for further analysis in naive, exposed, and HEV BDs, AHE patients, and CHE patients. No differences were found between exposed BDs and naive BDs, indicating that specific dysregulations were not sustained after clearance of HEV infection. In all HEV-infected groups, miR-122, miR-885, miR-194, and miR-30a were upregulated, while miR-221, miR-223, and miR-27a were downregulated, compared with naive BDs. In contrast, miR-335 was downregulated in AHE patients but not in HEV BDs and CHE patients compared with naive BDs. Although the role of these microRNAs in HEV infection has not yet been established, possible functions can be assigned based on previous studies describing their participation in other viral infections and cellular processes (30, 31).

Increased levels of miR-122 and miR-885 have commonly been related to liver damage. For instance, changes in the circulating levels of miR-122, which represents 70% of the total liver microRNAs (25), have previously been associated with liver diseases, including hepatocellular carcinoma, nonalcoholic steatohepatitis, and hepatitis C and E virus (HCV and HEV) infections (20, 24, 32–35). Similarly, an increase in the extracellular miR-885 abundance has been reported in patients with hepatopathies such as liver cirrhosis, hepatocellular carcinoma, acute HCV infection, and chronic hepatitis B (20, 36, 37). In our study, the circulating levels of miR-122 and miR-885 were significantly increased in all HEV-infected groups compared with naive BDs, indicating that HEV-mediated upregulation of these microRNAs occurs even in the absence of manifest liver injury. Although miR-122 failed to achieve statistical significance as a diagnostic test to discriminate asymptomatic HEV infections from those progressing to clinical disease, the positive correlation between ALT and miR-122 suggests that the latter could serve as a biomarker of liver damage during HEV infection. These results are consistent with previous studies that described a direct relationship between miR-122 levels and the clinical manifestations of viral hepatitis. For instance, circulating miR-122 levels were associated with hepatitis E severity during pregnancy (38) and correlated with ALT levels during HCV, HBV, and chronic HEV infections (20, 24, 26). Remarkably, miR-122 was proven essential for HCV replication because it stabilizes the viral genome, thereby increasing virus replication (39, 40). Because a similar role of miR-122 has been proposed in HEV replication (41), the miR-122 upregulation that we found in all HEV-infected groups could be part of the HEV mechanism to promote its replication, which is consistent with the positive correlation that we observed between miR-122 and plasma viral load.

The upregulation of miR-30a and miR-194 upon different types of viral infection has been associated with the modulation of antiviral immune response by negatively regulating type I interferon (IFN) signaling (42, 43). Interestingly, plasma exosomes from HCV and HCV+HEV BDs were enriched in miR-194, which is highly expressed in hepatocytes (44), suggesting that miR-194 expression can be altered upon both HCV and HEV infection (37). Although we analyzed total plasma microRNAs, which include microRNAs inside extracellular vesicles and free soluble microRNAs, our finding that circulating miR-194 was upregulated in all HEV-infected groups is consistent with an HEV-induced dysregulation of this microRNA to reduce type I IFN production.

Reduced expression of miR-221 was also observed during influenza A virus infection (45) and HCV infection (37, 46). As for miR-194 and miR-30a upregulation, miR-221 downregulation was associated with reduced type I IFN response and increased viral replication (45, 46).

On the other hand, miR-223 and miR-27a were reported to target cholesterol homeostasis and lipid metabolism, respectively (47, 48). The repression of miR-27a was associated with an increase in the cellular lipid content and HCV replication (48). In addition, miR-27a was found to negatively regulate the expression of several genes related to lipid metabolism such as *apolipoprotein E* (*APOE*), which may have an effect on HEV replication (49).Thus, the downregulation of miR-223 and miR-27a observed in all HEV-infected groups included in this study could be part of the HEV mechanism to improve viral replication by modulating lipid metabolism.

Furthermore, miR-335 was reported to play a critical role in the activation of inflammatory signaling pathways (50, 51). Because the main difference between asymptomatic HEV BDs and AHE patients is the downregulation of miR-335 in the latter, it is tempting to speculate that decreased levels of miR-335 during HEV infection could lead to manifest liver injury. Interestingly, a previous study found that exosomal miR-335 levels were dysregulated upon both HEV and HCV infections (37), being downregulated in HEV-infected patients but not in HEV BDs, which is in accordance with our observations.

Additionally, AHE patients in our study presented significantly higher levels of miR-122 and miR-194 and significantly lower levels of miR-221 and miR-27a than asymptomatic HEV BDs, suggesting that clinical manifestations of HEV infection could be associated with a larger HEV-induced alteration of the expression levels of microRNAs that participate in virus replication and antiviral immune response. Thus, along with reduced miR-335 levels, a higher dysregulation of miR-194 and miR-221 in AHE patients than in HEV BDs could induce a greater impairment of antiviral immune responses, thereby contributing to the different outcomes of HEV infection.

Notably, the plasma levels of miR-335, miR-194, and miR-221 were able to discriminate asymptomatic patients from those developing clinical symptoms. Circulating miR-335 levels showed the best specificity and sensitivity to distinguish between HEV BDs and AHE patients. In addition, they were also capable of discriminating acute infections from those progressing to a chronic stage despite the low number of samples available for these groups, which is a limitation of our study. Another limitation is that we have analyzed the microRNA profile during HEV genotype 3 infection; hence, it remains to be determined whether similar microRNA profiles would be observed for other HEV genotypes.

Symptomatic HEV infection in AHE patients was associated with miR-335 downregulation, which might lead to liver inflammation, along with a greater dysregulation of lipid metabolism through miR-223 and miR-27a and a greater impairment of type I IFN signaling through miR-194, miR-30a, and miR-221. Furthermore, we showed that the circulating levels of miR-335, miR-194, and miR-221 hold potential as predictive markers of HEV infection outcomes.

In summary, the current study demonstrates that HEV infection modifies the expression profile of host microRNAs that participate in lipid metabolism and type I IFN response in various types of viral infection, suggesting that HEV replication requires a fine regulation of both processes.

Our findings support the hypothesis that specific plasma microRNA signatures during HEV infection may lead to distinct regulation of antiviral immune response and virus replication, resulting in diverse clinical manifestations of HEV genotype 3 infection. The circulating microRNA signatures described in this study may improve our understanding of HEV pathogenesis and help in developing new treatments for severe HEV infections. Given the advantages of using the circulating microRNAs as noninvasive disease biomarkers, further investigation of the prognostic value of these microRNAs in a larger patient cohort is warranted.

## MATERIALS AND METHODS

### Selection and characterization of patient and blood donor samples.

This study was performed with plasma samples from patients diagnosed with either acute hepatitis E (AHE, *n* = 7) or chronic hepatitis E (CHE, *n* = 6) at the Vall d’Hebron Hospital (Barcelona, Spain), blood donors with asymptomatic acute HEV infection (HEV BDs, *n* = 9) identified by the routine HEV RNA screening at the Banc de Sang i Teixits (Barcelona, Spain), HEV RNA-negative blood donors with detectable anti-HEV IgG but undetectable anti-HEV IgM antibodies (exposed BDs, *n* = 10), and HEV RNA-negative blood donors without detectable anti-HEV IgG and IgM antibodies (naive BDs, *n* = 12). Plasma samples were kept at −30°C until testing.

The presence of anti-HEV IgG and IgM antibodies was assessed using the recomWell HEV IgG/IgM assay (Mikrogen Diagnostics). HEV RNA copies in plasma of infected patients were quantified at the Vall d’Hebron clinical laboratories with Cobas 6800 (Roche Diagnostics) and using a calibration curve based on the first World Health Organization International Standard for HEV RNA (Paul-Ehrlich-Institut code 6329/10), whereas viral loads in plasma of HEV BDs were determined at Banc de Sang i Teixits by in-house reverse transcription-PCR (RT-PCR) as previously described (52). All HEV isolates belonged to genotype 3 according to the genotyping method described by Bes et al. (53). Plasma levels of alanine transaminase (ALT), aspartate transaminase (AST), and gamma-glutamyl transpeptidase (GGT) in HEV-infected patients and HEV BDs were analyzed at the Vall d’Hebron clinical laboratories or at a reference biochemistry laboratory, respectively. Data from serological and biochemical analysis, along with demographic, clinical, and virological information recorded before sample anonymization, are shown in Table 1.

**TABLE 1:** Demographic, clinical, and virological characteristics of HEV-infected patients and blood donors^*a*^

ID	Gender (M/F)	Age (yr)	Log_10_ viral load (IU/mL)	Anti-HEV IgM/IgG	ALT (IU/mL)	AST (IU/mL)	Bilirubin (mg/dL)	Immunosuppression/cause	Ribavirin therapy/response^*b*^
AHE-1	F	52	3.0	+/+	111	46	0.73	None	No
AHE-2	M	59	5.8	+/+	1,243	308	1.3	None	No
AHE-3	M	53	5.5	+/+	457	129	7.57	None	No
AHE-4	M	42	1.6	−/−	25	27	0.59	None	No
AHE-5	M	45	5.4	+/+	1,534	1,168	4.55	None	No
AHE-6	M	50	2.9	+/+	40	24	1.48	None	No
AHE-7	M	48	DNQ	+/+	1,535	3,213	19.83	None	No
HEV BDs-1	F	45	3.0	+/+	43	80	0.34	None	No
HEV BDs-2	F	21	3.7	+/+	161	58	0.78	None	No
HEV BDs-3	M	64	3.5	+/+	60	30	0.48	None	No
HEV BDs-4	M	28	3.1	−/−	88	83	0.54	None	No
HEV BDs-5	M	48	1.6	+/+	45	22	0.33	None	No
HEV BDs-6	M	57	1.6	−/−	21	27	0.44	None	No
HEV BDs-7	M	59	4.2	+/+	97	39	0.32	None	No
HEV BDs-8	M	40	2.4	+/+	29	28	0.50	None	No
HEV BDs-9	M	60	1.6	+/+	46	36	0.51	None	No
CHE-1	M	62	DNQ	+/+	29	26	0.57	Yes/renal transplant	Yes/virological response
CHE-2	F	41	DNQ	+/+	16	20	0.36	Yes/lung transplant	Yes/partial response
CHE-3	M	68	DNQ	+/+	90	90	0.82	Yes/renal and lung transplant	Yes/relapse
CHE-4	M	23	4.6	+/+	42	36	0.56	Yes/renal transplant	Yes/partial response
CHE-5	F	52	6.7	+/+	66	69	6.23	Yes/renal transplant	Yes/virological response
CHE-6	F	32	6.1	+/+	152	128	0.48	Yes/anti-TNF therapy and primary immunodeficiency	Yes/virological response

a ID, identifier; M/F, male/female; DNQ, detected but not quantified; ALT, alanine transaminase; AST, aspartate transaminase; GGT, gamma-glutamyl transpeptidase; AHE, acute hepatitis E patients; HEV BDs, HEV-infected blood donors; CHE, chronic hepatitis E patients; NA, not applicable; TNF, tumor necrosis factor.

b Definitions of ribavirin therapy responses: virological response, undetectable HEV-RNA at end of therapy and 12 months after; partial response, 2-log decrease of HEV-RNA during therapy but inability to achieve undetectable viral load; relapse, HEV-RNA undetectable at the end of therapy but detectable during follow-up.

This study was conducted in accordance with the 1964 Declaration of Helsinki and was approved by the Vall d’Hebron ethics committee [project code PR(BST)351/2017].

### Plasma microRNA RT-qPCR profiling.

To identify potentially HEV-regulated miRNAs, RNA samples from 200 μL plasma of 6 AHE patients and 6 naive BDs were isolated using the miRNeasy Serum/Plasma Advanced kit (Qiagen), reverse transcribed with the miRCURY LNA RT kit (Qiagen), and run on the miRCURY LNA miRNA PCR Serum/Plasma Focus panel (Qiagen) using miRCURY LNA SYBR green master mix (Qiagen). The experiments were performed at Qiagen Genomic Services (Hilden, Germany). An RNA spike-in mixture kit (Qiagen) was added prior to RNA isolation according to the quality data control pipeline of Genomic Services. RNA spike-ins UniSp2, UniSp4, and UniSp5 were used to evaluate the RNA extraction efficiency, and UniSp6 was added as a cDNA synthesis control. In addition, the DNA spike-in UniSp3 was used to assess potential quantitative PCR (qPCR) inhibitions. Hemolysis was assessed by the ratio between miR-451, which is expressed in red blood cells, and miR-23a, which is relatively stable in plasma. The quantification cycle (*C_q_*) values were normalized based on the global mean (*C_q_* mean for the assays detected in all samples) because its stability was found to be higher than that of any single microRNA in the data set as measured by the NormFinder algorithm (54). The delta *C_q_* values (d*C_q_*) obtained after normalization of data were used to calculate the differences in expression levels (dd*C_q_*), which were converted to fold change with the formula 2^dd^*^Cq^*. Eight differentially expressed microRNAs were selected for further characterization of specific microRNA dysregulations during HEV infection.

### Characterization of specific microRNA signatures.

The microRNAs analyzed in this study were among the uppermost differentially expressed miRNAs in the profiling assay and include miR-122-5p, miR-194-5p, miR-221-3p, miR-223-3p, miR-27a-3p, miR-30a-5p, miR-335-5p, and miR-885-5p. The microRNAs miR-222-3p and let-7i-5p were selected as endogenous microRNA controls because they showed the highest stability values in the profiling experiment. RNA from 32 additional samples, including 9 HEV BDs, 1 AHE patient, 6 CHE patients, 6 naive BDs, and 10 exposed BDs, was extracted and processed as described above. The expression levels of the selected microRNAs were assessed using a miRCURY LNA miRNA PCR custom panel. The experimental procedure was also performed at Qiagen Genomic Services (Hilden, Germany). Data were normalized to the average from miR-222-3p and miR-let-7i-5p assays. RNA and DNA spike-ins and hemolysis controls were applied as described above.

### Statistical analysis.

Principal-component analysis (PCA) was performed on all samples and the top 50 miRNAs with the largest variation across all samples. The normal distribution of the normalized *C_q_* data was assessed by a Shapiro-Wilk normality test. Then, different microRNA expression between two groups was assessed by unpaired two-tailed *t* test for normally distributed data or two-tailed Mann-Whitney test (MW) for nonparametric data. *P* value and *P* value adjusted for multiple testing by the Benjamini-Hochberg correction (*P*_BH_) were used to identify dysregulated microRNAs. A volcano plot was constructed with the *P* values and the fold change data of AHE patients versus naive BDs of all analyzed microRNAs. Receiver operating characteristic (ROC) plot analysis was performed with the normalized *C_q_* values to calculate the area under the curve (AUC), statistical significance, sensitivity, and specificity of microRNAs to predict the clinical outcome of HEV infection. The correlations between miR-122 and ALT and between miR-122 and viral load were calculated with nonparametric Spearman correlation and Pearson correlation coefficients, respectively. Statistical analyses were performed using GraphPad Prism 8.4.3 software (GraphPad Software, San Diego, CA). *P* values of <0.05 were considered statistically significant.
