# Supplementary material for: Specific Plasma MicroRNA Signatures Underlying the Clinical Outcomes of Hepatitis E Virus Infection
Source: Microbiol Spectr. 2023 Jan 25;11(1):e04664-22. doi: 10.1128/spectrum.04664-22 (PMC9927377; doi:10.1128/spectrum.04664-22)
Supplement: Supplemental file 1 — Table S1 and Fig. S1 to S4. Download spectrum.04664-22-s0001.pdf, PDF file, 0.7 MB [file spectrum.04664-22-s0001.pdf]

Supplementary Table 1. Data summary of differentially expressed miRNAs that passed the Benjamini-Hochberg multiple comparison correction. The last two columns show the p-value from the t-test and the Benjamini-Hochberg adjusted p-value (P_{BH}).

microRNA	AHE (SD)	Naïve BDs (SD)	dCq AHE (mean)	dCq Naïve BDs (mean)	ddCq AHE - Naïve	Fold change	p-value	P_{BH}
hsa-miR-27a-3p	0,32	0,29	1,60	2,89	-1,29	-2,45	0,00003	0,003
hsa-miR-23a-3p	0,24	0,21	2,71	3,64	-0,93	-1,90	0,00004	0,003
hsa-miR-24-3p	0,36	0,22	2,34	3,56	-1,22	-2,34	0,00008	0,003
hsa-miR-335-5p	0,50	0,59	-3,54	-1,51	-2,02	-4,06	0,00008	0,003
hsa-miR-223-3p	0,49	0,18	3,89	5,77	-1,88	-3,68	0,00009	0,003
hsa-miR-194-5p	0,91	0,80	-0,49	-3,51	3,02	8,10	0,00012	0,003
hsa-miR-155-5p	0,31	0,31	-4,23	-6,70	2,47	5,53	0,00025	0,005
hsa-miR-103a-3p	0,21	0,17	1,63	1,00	0,62	1,54	0,00025	0,005
hsa-miR-15b-5p	0,33	0,11	1,43	2,51	-1,08	-2,11	0,00027	0,005
hsa-miR-122-5p	1,75	0,53	1,92	-3,25	5,17	35,89	0,00048	0,008
hsa-miR-107	0,24	0,28	1,31	0,56	0,75	1,68	0,00061	0,009
hsa-miR-885-5p	1,70	0,69	-2,22	-6,76	4,53	23,13	0,00079	0,010
hsa-miR-148b-3p	0,62	0,41	-1,28	0,24	-1,52	-2,86	0,00080	0,010
hsa-miR-221-3p	0,51	0,66	0,84	2,47	-1,63	-3,09	0,00088	0,010
hsa-miR-142-5p	0,44	0,29	0,48	1,53	-1,05	-2,07	0,00094	0,010
hsa-miR-28-3p	0,24	0,49	-3,39	-2,23	-1,17	-2,24	0,00103	0,011
hsa-miR-152-3p	0,30	0,33	-1,16	-0,34	-0,82	-1,76	0,00116	0,012
hsa-miR-376a-3p	0,67	1,03	-2,61	-0,25	-2,36	-5,13	0,00127	0,012
hsa-miR-92a-3p	0,26	0,29	4,85	4,16	0,70	1,62	0,00139	0,012
hsa-miR-30a-5p	1,00	0,69	-1,18	-3,44	2,26	4,81	0,00143	0,012
hsa-let-7d-3p	0,50	0,26	-1,72	-0,59	-1,13	-2,19	0,00147	0,012
hsa-miR-199a-3p	0,47	0,79	0,20	1,95	-1,75	-3,36	0,00154	0,012
hsa-miR-215-5p	1,20	0,53	-1,49	-4,16	2,67	6,38	0,00163	0,012
hsa-miR-142-3p	0,73	0,65	2,40	0,72	1,67	3,19	0,00189	0,013
hsa-miR-143-3p	1,10	0,40	-3,84	-0,78	-3,06	-8,34	0,00218	0,014
hsa-miR-192-5p	1,52	1,15	-0,78	-4,03	3,25	9,48	0,00223	0,014
hsa-miR-19b-3p	0,32	0,12	3,04	3,71	-0,67	-1,59	0,00251	0,015
hsa-miR-20b-5p	0,93	0,15	-5,69	-8,34	2,64	6,25	0,00262	0,015
hsa-miR-144-3p	0,48	0,82	3,20	1,55	1,66	3,15	0,00266	0,015
hsa-let-7b-5p	0,26	0,32	1,39	0,72	0,67	1,59	0,00273	0,015
hsa-miR-10b-5p	0,97	0,87	-1,77	-4,14	2,38	5,19	0,00273	0,015
hsa-miR-27b-3p	0,31	0,51	0,70	1,70	-1,00	-2,01	0,00321	0,017
hsa-miR-205-5p	0,72	0,48	-4,32	-5,97	1,66	3,15	0,00362	0,018
hsa-miR-423-3p	0,96	0,45	-0,84	0,98	-1,82	-3,53	0,00376	0,019
hsa-miR-652-3p	0,43	0,42	-1,67	-0,80	-0,87	-1,83	0,00521	0,025
hsa-miR-1260a	0,88	0,21	0,37	-1,26	1,63	3,09	0,00529	0,025
hsa-miR-376c-3p	0,56	1,19	-2,35	-0,34	-2,02	-4,05	0,00692	0,031
hsa-miR-451a	0,57	0,92	7,32	5,76	1,56	2,96	0,00723	0,031
hsa-miR-154-5p	0,34	1,01	-5,18	-3,43	-1,74	-3,35	0,00725	0,031
hsa-miR-34a-5p	1,62	0,45	-1,72	-5,08	3,36	1,03	0,00762	0,031
hsa-miR-150-5p	0,82	0,85	1,19	-0,41	1,60	3,03	0,00769	0,031
hsa-miR-423-5p	0,37	0,30	0,97	1,63	-0,66	-1,58	0,00779	0,031
hsa-miR-99a-5p	1,02	0,40	-1,46	-3,13	1,67	3,19	0,00824	0,032
hsa-let-7a-5p	0,30	0,52	0,74	-0,11	0,85	1,80	0,00836	0,032

hsa-miR-590-5p	0,56	0,38	-2,66	-1,73	-0,92	-1,90	0,00866	0,032
hsa-miR-130a-3p	0,56	0,68	1,35	2,53	-1,18	-2,26	0,00870	0,032
hsa-miR-146a-5p	0,53	0,84	0,79	2,16	-1,36	-2,57	0,00891	0,032
hsa-miR-7-5p	1,09	0,86	-4,09	-5,84	1,75	3,37	0,01203	0,042
hsa-miR-197-3p	0,82	0,16	-2,27	-1,03	-1,24	-2,37	0,01272	0,044
hsa-miR-25-3p	0,57	0,37	2,63	1,77	0,86	1,81	0,01395	0,047
hsa-miR-23b-3p	0,52	0,21	0,92	1,67	-0,75	-1,69	0,01416	0,047

4

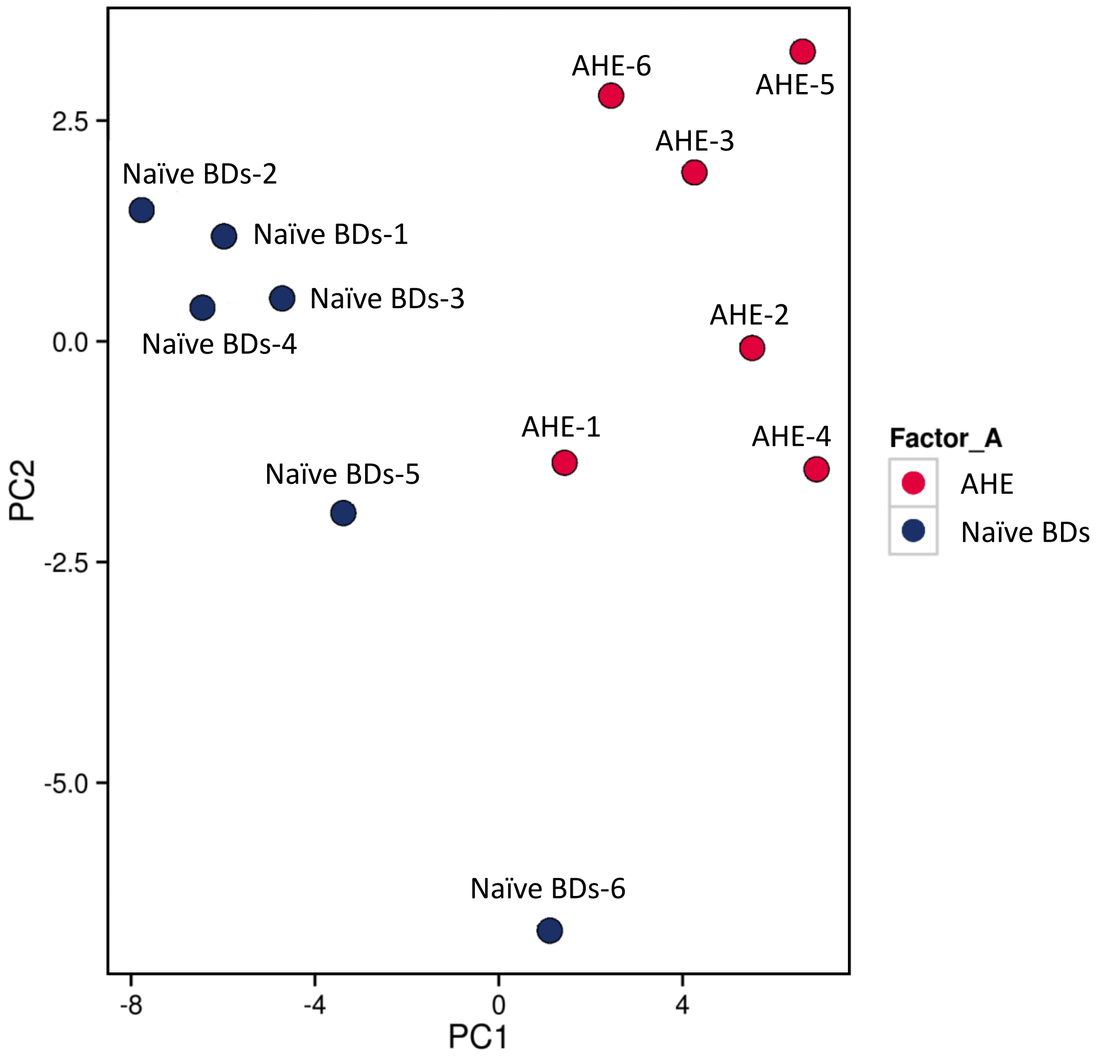

Supplementary Figure 1. Principal Component Analysis (PCA) showing that samples from HEV-infected patients and non-infected controls were naturally separated based on the microRNA expression profile. The PCA was performed on all samples and on the top 50 miRNAs with the largest variation across all samples. dCq values were used for the analysis. AHE, acute hepatitis E; Naïve BDs, naïve blood donors.

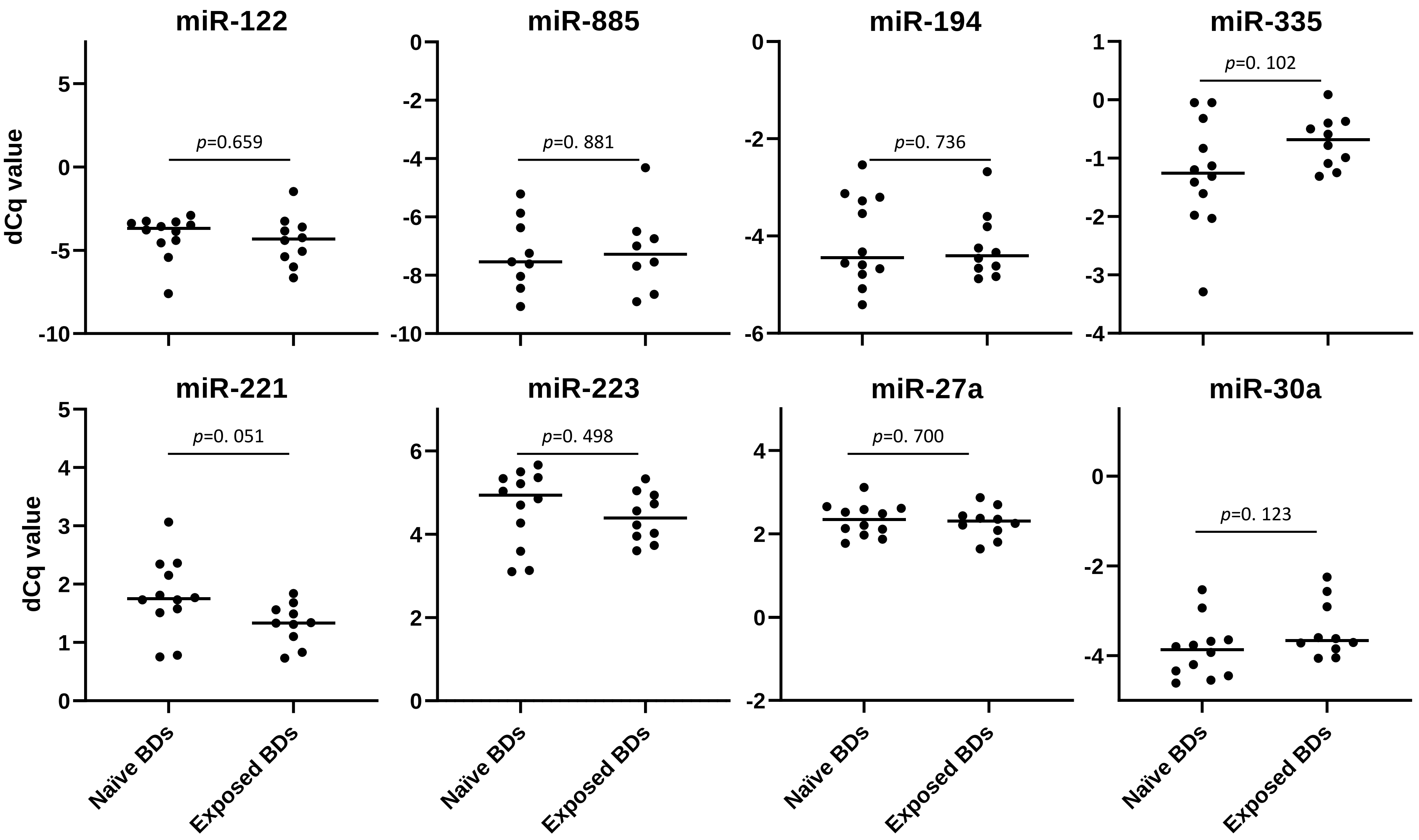

Supplementary Figure 2. HEV infection does not result in a persistent deregulation of investigated microRNAs after viral clearance. dCq values of miR-122, miR-885, miR-194, miR-335, miR-221, miR-223, miR-27a, and miR-30a in plasma of Naïve BDs (n = 12) and Exposed BDs (n = 10). Each dot represents an individual sample. P-values between Naïve and Exposed BDs were analyzed by unpaired two-tailed t-test. Naïve BDs, naïve blood donors (HEV RNA-negative blood donors without detectable anti-HEV antibodies); Exposed BDs, exposed blood donors (HEV RNA-negative blood donors with detectable anti-HEV antibodies).

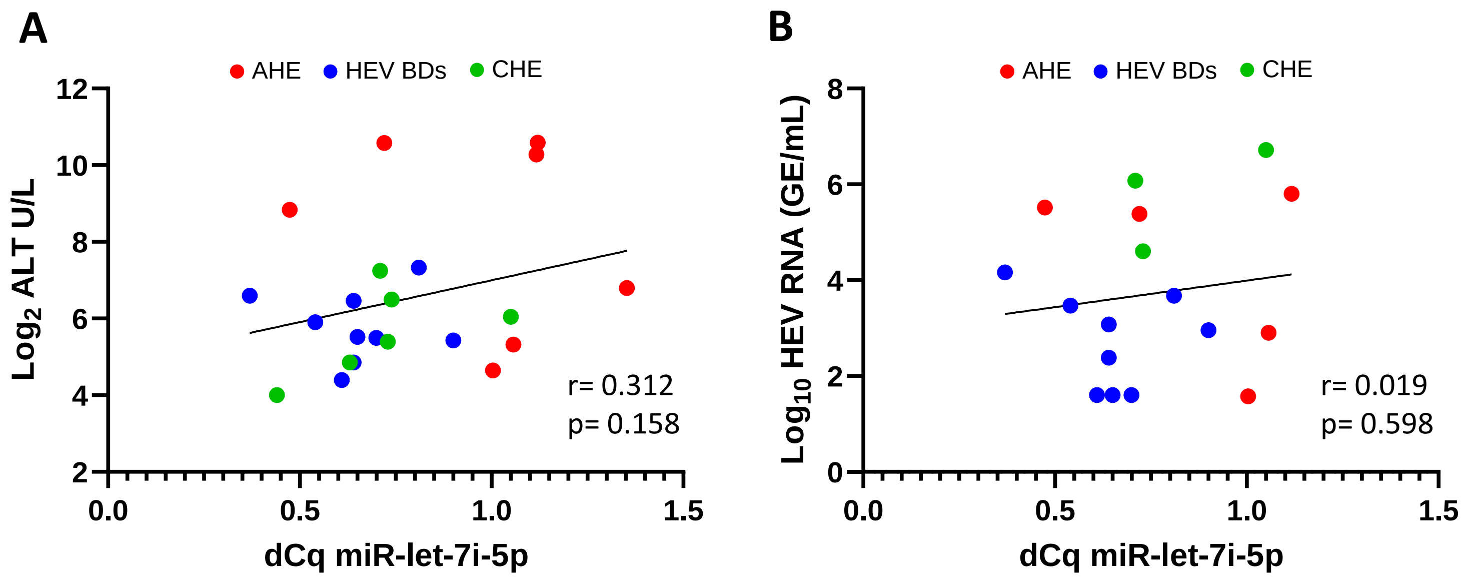

Supplementary Figure 3. Circulating levels of miR-let-7i-5p did not correlate with ALT levels and viral load. The degree of association between miR-let-7i-5p and ALT (A) and between miR-let-7i-5p and viral load (B) was calculated by Spearman rank correlation and Pearson correlation coefficients, respectively. Correlation coefficients (r) and p-values are indicated in the graphs. AHE, acute hepatitis E; HEV BDs, HEV-infected blood donors; CHE, chronic hepatitis E.

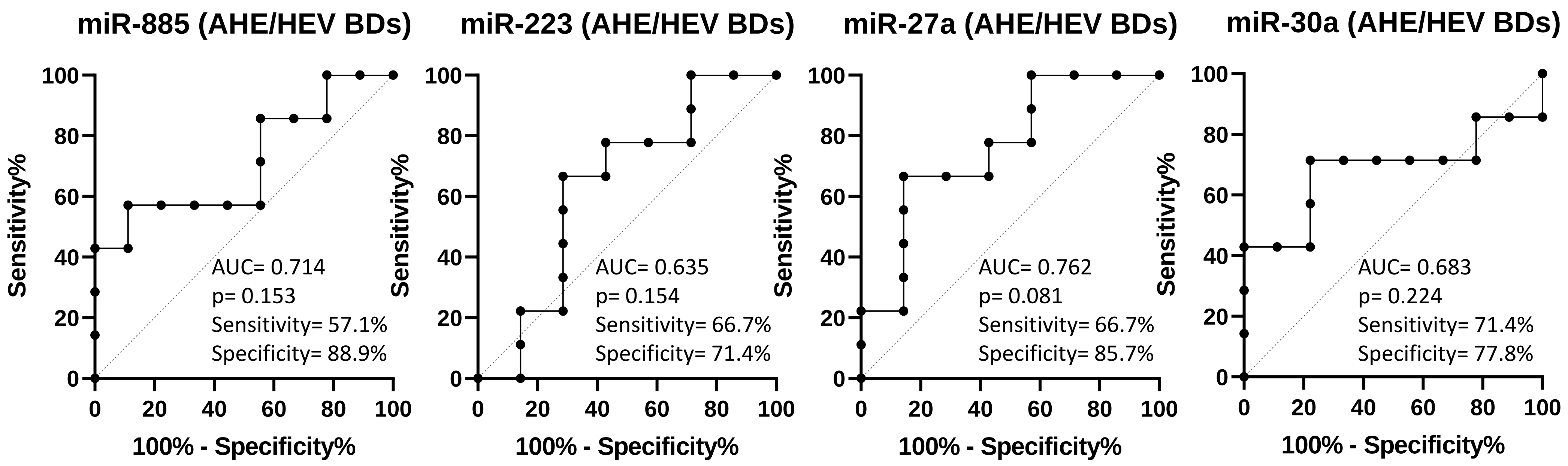

Supplementary Figure 4. Circulating levels of miR-885, miR-223, miR-27a, and miR-30a do not distinguish between AHE and HEV BDs. ROC plot analyses of predictive discrimination of AHE from HEV BDs with AUC, p-value, sensitivity, and specificity values. P-values < 0.05 were considered significant. Sensitivity and specificity values are given based on the best compromise between these parameters.
